# Co-Expression Network and Time-Course Expression Analyses to Identify Silk Protein Regulatory Factors in *Bombyx mori*

**DOI:** 10.3390/insects13020131

**Published:** 2022-01-26

**Authors:** Yudai Masuoka, Wei Cao, Akiya Jouraku, Hiroki Sakai, Hideki Sezutsu, Kakeru Yokoi

**Affiliations:** 1 Insect Design Technology Module, Division of Insect Advanced Technology, Institute of Agrobiological Sciences, National Agriculture and Food Research Organization (NARO), 1-2 Owashi, Tsukuba 305-8634, Ibaraki, Japan; joraku@affrc.go.jp; 2 Research Center for Agricultural Information Technology (RCAIT), National Agriculture and Food Research Organization (NARO), 1-31-1 Kannondai, Tsukuba 305-0856, Ibaraki, Japan; sou197@affrc.go.jp; 3 Silkworm Research Module, Division of Silk-Producing Insect Biotechnology, Institute of Agrobiological Sciences, National Agriculture and Food Research Organization (NARO), 1-2 Owashi, Tsukuba 305-8634, Ibaraki, Japan; sakaih786@affrc.go.jp (H.S.); hsezutsu@affrc.go.jp (H.S.)

**Keywords:** co-expression network analysis, *Bombyx mori*, silk protein, sericin, fibroin, transcription factor

## Abstract

**Simple Summary:**

Previous studies have reported how the silk production ability of *Bombyx mori* can be enhanced, but the mechanism that regulates silk protein genes remains unclear. We performed co-expression network analysis using *networkz*, an in-house program, which led to the identification of 91 transcription factors were co-expressed with silk protein genes. Of them, 13 transcripts were identified to be novel regulatory factors by time-course expression analysis during the fifth instar larvae stage. Their expression patterns were highly relevant to those of silk protein genes. Our results suggest that the two-step expression screening was robust and highly sensitive to screen relative genes, and a complex mechanism regulates silk protein production in *B. mori*. The novel candidates that were identified herein can serve as key genes to develop methods to enhance the silk protein production ability of *B. mori*.

**Abstract:**

*Bombyx mori* is an important economic insect and an animal model in pharmacomedical research. Although its physiology has been studied for many years, the mechanism via which silk protein genes are regulated remains unclear. In this study, we performed two-step expression screening, namely co-expression network and time-course expression analyses to screen silk protein regulation factors. A co-expression network analysis using RNA-seq data that were obtained from various tissues, including the silk glands of *B. mori*, was performed to identify novel silk protein regulatory factors. Overall, 91 transcription factors, including some known ones, were found to be co-expressed with silk protein genes. Furthermore, time-course expression analysis during the fifth instar larvae stage revealed that the expression pattern of 13 novel transcription factors was highly relevant to that of silk protein genes and their known regulatory factor genes. In particular, the expression peak of several transcription factors (TFs) was detected before the expression of silk protein genes peak. These results indicated that a larger number of genes than expected may be involved in silk protein regulation in *B. mori*. Functional analyses of function-unknown transcription factors should enhance our understanding of this system.

## 1. Introduction

Silkworms (*Bombyx mori*) generate silk proteins; they are an economically important insect in sericulture and have proved their value in biotechnology as a bioreactor for the production of recombinant proteins and silk-based biomaterials. Silk proteins can be broadly classified into sericin and fibroin, which are secreted from the middle and posterior silk glands (SGs), respectively. The SG consists of endomitotic cells [1], and the expression of genes encoding these proteins shows a considerably increase in the fifth (last) instar larvae stage. The elucidation of mechanisms that regulate the expression of such genes is necessary to further enhance the ability of this insect to produce silk.

In previous studies, it has been reported that some transcription factors (TFs), including homeobox genes, regulate the expression of silk protein genes [2,3]. For instance, *Antennapedia* (*Antp*), a Hox gene that controls leg formation, directly regulates the expression of *sericin1* in the middle SG [4,5]. Further, silk gland factor-2 (SGF2), a protein complex containing the homeodomain protein Arrowhead (Awh), LIM domain-binding protein, and sequence-specific single-stranded DNA-binding protein, evidently regulates the expression of genes encoding fibroin in the posterior SG [6,7]. The *silk gland factor-1* (*SGF1*), containing a forkhead domain, and *silk gland factor-3* (*SGF3*) genes are involved in regulating sericin1 expression [8,9,10]. Besides, sage, encoding a basic helix-loop-helix TF, is involved in regulating the expression of fibroin heavy-chain along with *SGF1* [11].

Although some genes have been identified to function as expression regulators of silk protein genes, the pertinent regulatory mechanism and pathways still remain unclear. Furthermore, these regulatory factors, such as hox genes, have been known to possess other functions [3] which can lead to lethal effects when they are genetically modified. To avoid the risk as much as possible, the factors that are specific to the silk gene regulation in the silk gland are desirable as targets for genetic modification to increase silk yield. Thus, a co-expression relationship among silk proteins and their regulatory genes (known and unknown) needs to be elucidated. For this purpose, gene expression network analysis using large-scale transcriptome data is essential. Co-expression network analysis is an effective approach to elucidate groups of genes that are showing distinct co-expression patterns among phenotypes. This approach has been widely adopted for various purposes, including to predict diseases in humans [12], detect metabolic pathways involving organic compounds and stress-responsive genes in plants [13,14,15], and determine gene sets that are related to biological processes in bacteria [16]. In insects, co-expression network analysis has been mainly used in model species considering the availability of abundant transcriptome data. Co-expressed genes at different stages, including young lncRNA genes, have been detected in *Drosophila melanogaster* [17]. In mosquitoes (*Aedes aegypti*), infection-responsive genes were identified using genome-wide transcriptome profiling, including co-expression network analysis [18]. In *B. mori*, lncRNA and domestication-related genes including silk gland-related genes were identified by co-expression network analysis [19,20]. Although co-expression network analysis is actually useful for identifying relevant gene groups, further detailed analysis, such as time-course expression analysis, is necessary to detect more important genes. Functional analysis of screened candidates is thus required to understand the mechanisms regulating silk protein genes.

Herein we attempted to identify genes regulating the expression of silk protein genes using co-expression network as well as time-course expression analyses. Screening precision is dependent on the input data volume and variation, and standard Java-based tools that are used in co-expression network analysis (e.g., Gephi and Cytoscape) take a long time to process large quantities of expression data. Accordingly, we developed a fast C++-based tool to quickly process large expression datasets. Co-expression network analysis was performed using published transcriptome data [21,22,23,24] comprising five SG regions [anterior SG (ASG), anterior-middle SG (A-MSG), middle-middle SG (M-MSG), posterior-middle SG (P-MSG), and posterior SG (PSG)], Malpighian tubule (MT), testis (TT), and ovary (OV). A total of six silk protein genes [*sericin1*, *sericin2*, *sericin3*, *fibroin heavy-chain* (*fibroin-H*), *fibroin light-chain* (*fibroin-L*), and *fibrohexamerin* (*P25*)] were selected as target genes to search for regulatory factors. There were also five existing regulatory genes [*SGF1*, *SGF3*, *sage*, *Antp*, and *Awh* (main isoform PA)] that were also chosen as target genes. TFs that showed expression patterns that were similar to those of the target genes were subjected to time-course expression analysis, which was performed at A-MSG, M-MSG, P-MSG, and PSG on every day during last instar larva (day zero to seven). Further, TFs with expression patterns that were related to those of target genes were shortlisted as candidates of silk protein regulatory genes. Our results provide insights into how silk protein genes are regulated; moreover, the genes that are discussed herein can be used as targets to improve silk protein production ability.

## 2. Materials and Methods

### 2.1. Constructing a Gene Co-Expression Network and Detecting Modules

We developed a command line tool named networkz to handle large gene co-expression datasets (or gene expression profiles) and to perform co-expression network analysis. networkz was written in C++ and the source code is available at https://github.com/davecao/networkz.git (accessed on 23 December 2021); it is based on Boost Graph Library v1.70 [https://www.boost.org (accessed on 23 December 2021)] for graph data structure operations and Eigen Library v3.3.90 [(https://gitlab.com/libeigen/eigen/-/releases (accessed on 23 December 2021)] for matrix operations.

The relationships among genes in the co-expression dataset can be represented by a network, which is an undirected and weighted graph consisting of vertices and edges; herein genes are referred to as vertices while their edges represent the pairwise co-expression measure. To construct an initial co-expression network, we selected a significance measure threshold to determine the connected gene pairs with a significant co-expression relationship, and then modules (or hub genes) that were highly connected with others were detected in the subsequent analysis.

In this study, a gene profile is denoted as a vector with *m* components; $x_{i}=\left(x_{i,1},x_{i,2},\ldots ,x_{i,m}\right).\;$ Then *n* gene expression profiles were represented by an *n* × *m* matrix; $X={\left(x_{1},x_{2}\ldots ,\;x_{n}\right)}^{T}$. The expression measure between the genes p and q ($d_{p,q}$,) was defined as follows:

$$\begin{array}{c} d_{p,q}=1-\left|corr\left(p,q\right)\right|\\ corr\left(p,q\right)=corr\left(x_{i},x_{j}\right)=\frac{\sum_{k=1}^{m}\left(x_{i,k}-\bar{x_{i}}\right)\left(x_{j,k}-\bar{x_{j}}\right)}{\sqrt{\sum_{k=1}^{m}{\left(x_{i,k}-\bar{x_{i}}\right)}^{2}\sum_{k=1}^{m}{\left(x_{j,k}-\bar{x_{j}}\right)}^{2}}},\;i,\;j=1,\;\ldots ,\;n,\;i\neq j \end{array}$$

wherein |corr(p,q)| represents the absolute value of Pearson’s correlation coefficient between the expression profiles of p and q; $\bar{x_{i\;}}\;$ and $\bar{x_{j\;}}$ present mean of $x_{i}\;\mathrm{and}\;x_{j}$, respectively. The smaller the value of $d_{p,q}$ is, the higher the likelihood of the two genes (p and q) in the network being interconnected (i.e., showing high correlation in terms of pairwise gene similarity). The threshold of 0.1 was selected via trial and error.

To detect modules in the initially constructed network, we further employed the Kruskal’s algorithm [25], as vertices were much more than edges, to find a minimum spanning tree (MST) with minimum sum of edge weights; then, the Louvain method [26] was performed on the MST to assign each gene with a community ID. Finally, modules of interest were found.

### 2.2. Co-Expression Network Analysis

For co-expression network analysis with *networkz*, we used transcript-level transcripts per million (TPM) values as expression data of two RNA-seq data series, which were used for the assembly and verification of the current reference transcriptome dataset of *B. mori* in our previous study [24] The first RNA-seq data series (SRA Run ID: DRR068893-068895 and DRR095105-095116) was obtained from the fat body (FB), midgut (MG), MT, whole SG (SG), and TT of the o751 strain last instar larvae on third day (Table 1) [21,22,23]. The second RNA-seq data series (SRA Run ID: DRR186474-186503) was obtained from the aforementioned five SG regions (ASG, A-MSG, M-MSG, P-MSG, and PSG), FB, MG, MT, TT, and OV of p50T strain last instar larvae on third day (Table 1) [24]. The transcript-level TPM expression data that were used in this study are available at “expression data of each transcript in multiple tissues” in the study by Yokoi et al. 2021 (doi: 10.18908/lsdba.nbdc02443-002.V001), in which 51,926 transcripts were used as reference sequences for TPM calculation [24]. Herein we used the same transcript ID as that of reference transcript sequences. The silk protein genes (*sericin1*, *sericin2*, *sericin3*, *fibroin-H*, *fibroin-L*, and *P25*) and five existing regulatory genes (*SGF1*, *SGF3*, *sage*, *Antp*, and *Awh*) served as target genes. Target network modules containing transcripts (isoforms) of the target genes were identified from network modules that were constructed by *networkz*. As the target genes showed multiple isoforms, multiple target network modules were identified for each target gene. The transcripts that were annotated with major TF-specific motif in target network modules were screened as candidate TFs.

### 2.3. RNA Extraction

To extract total RNA, fifth instar larva of the w-1 pnd strain of *B. mori* were kept on an artificial diet (Nihon Nosan Kogyo, Yokohama, Japan) at 25 °C under LD 12:12 h. The SGs of three male and female insects were then extracted every day during the last instar period (day 0–7). Total RNA was isolated from one pair of SGs for each individual using TRIzol (Invitrogen, Carlsbad, CA, USA) and RNeasy Plus Mini Kit (Qiagen, Hilden, Germany), and the wet weight of the whole SG was measured using the other pair of SG.

### 2.4. Gene Expression Analysis

For quantitative RT-PCR (qRT-PCR), cDNAs were synthesized from 500 ng RNA using the Prime Script^®^ RT reagent kit (Takara, Tokyo, Japan). *Elongation factor-2* (*EF-2*) was used as a reference gene to calculate the relative expression levels [27,28]. Except EF-2, the specific primers were newly designed for each gene using Primer3Plus (Table S1) [29]. The expression levels of each gene were quantified using TB Green™ Premix Ex Taq™ II (Takara, Tokyo, Japan) on a Light Cycler 480 (Roche Diagnostics, Mannheim, Germany). Biological triplicates were subjected to qRT-PCR, and each sample contained cDNA from each tissue of a male and female pair. The relative expression levels of each gene were calculated by adopting the standard curve method. Statistical analysis was performed using ANOVA and the Tukey–Kramer test for comparisons among the last instar period. These statistical analyses were performed using the statistical software Mac Statistical Analysis ver. 2.0.

## 3. Results

### 3.1. Co-Expression Network Analysis with Tissue Expression Data

**Figure 1:**
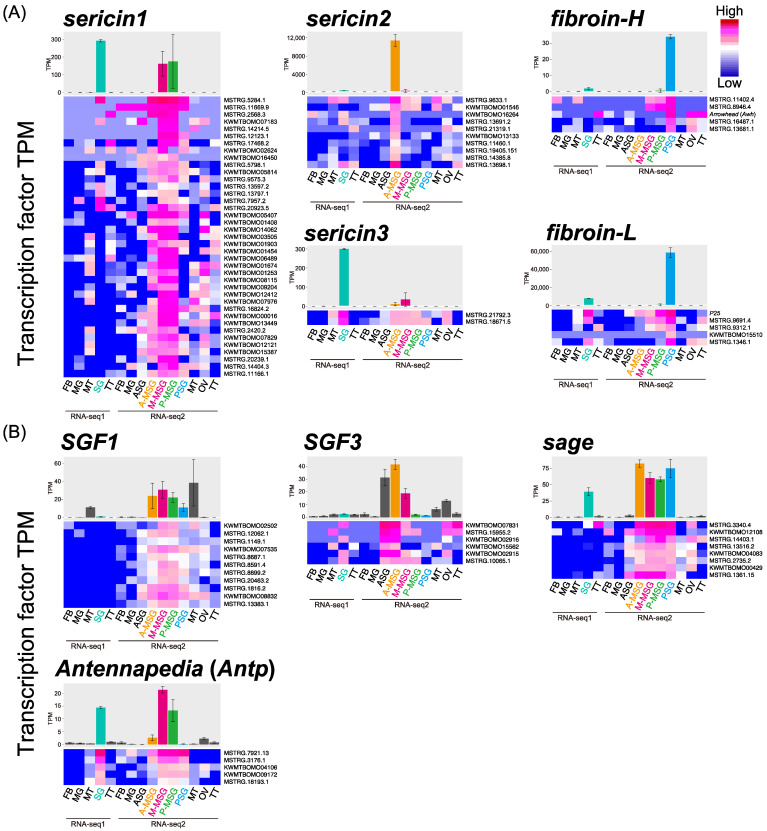
TPM (mean ± SE, biological triplicates) of silk protein genes (**A**) and TFs (**B**) from RNA-seq analysis and heatmap that was based on TPM of each module gene. The *sericin1* and *sericin2* graphs were drawn based on TPM values of main transcripts (*sericin1*: *KWMTBOMO06216*, *sericin2*: *KWMTBOMO06334*). Transcript ID is indicated on the right. Tissues that were used for RNA-seq are indicated under the heatmap.

Co-expression network analysis was performed with *networkz* to detect the candidate genes that regulate silk protein genes or the known regulatory factors of silk proteins. The program (*networkz*) allocates each transcript to the single most plausible network module. In total, 1022 network modules were generated, and the transcripts of the target genes were identified in 20 network modules (Table 2, Data S1). Of these, two target genes, *P25* and *Awh*, belonged to the *fibroin-L* and *fibroin-H* modules, respectively, whereas four known TFs (*SGF1*, *SGF3*, *sage*, and *Antp*) were sorted into different modules. Overall, 91 TFs were detected in the above 20 modules. The sericin1 modules, which showed a specific expression pattern in the M-MSG and P-MSG, contained 39 TFs among 565 transcripts. In addition, the sericin2 modules, which showed a specific expression pattern in the A-MSG, contained 11 TFs among 289 transcripts, and the sericin3 module, which showed a specific expression pattern in the whole SG of RNA-seq data-1 and M-MSG, contained two TFs among 36 transcripts (Figure 1A, Table 2). Although *fibroin-H* and *fibroin-L* showed PSG-specific expression patterns, they were separated into different network modules because of differences in TPM values. These modules contained nine TFs among 122 transcripts (Figure 1A, Table 2). The modules of four known TFs (*SGF1*, *SGF3*, *sage*, and *Antp*) contained >100 transcripts, including 5–11 TFs (Figure 1B, Table 2). All the obtained TFs were similarly expressed at one or more tissues with each target gene. Collectively, 91 transcripts were screened as candidate TFs that seem to regulate target gene expression.

### 3.2. Time-Course Expression Analysis of the Four SG Regions during the Last Instar Period

**Figure 2:**
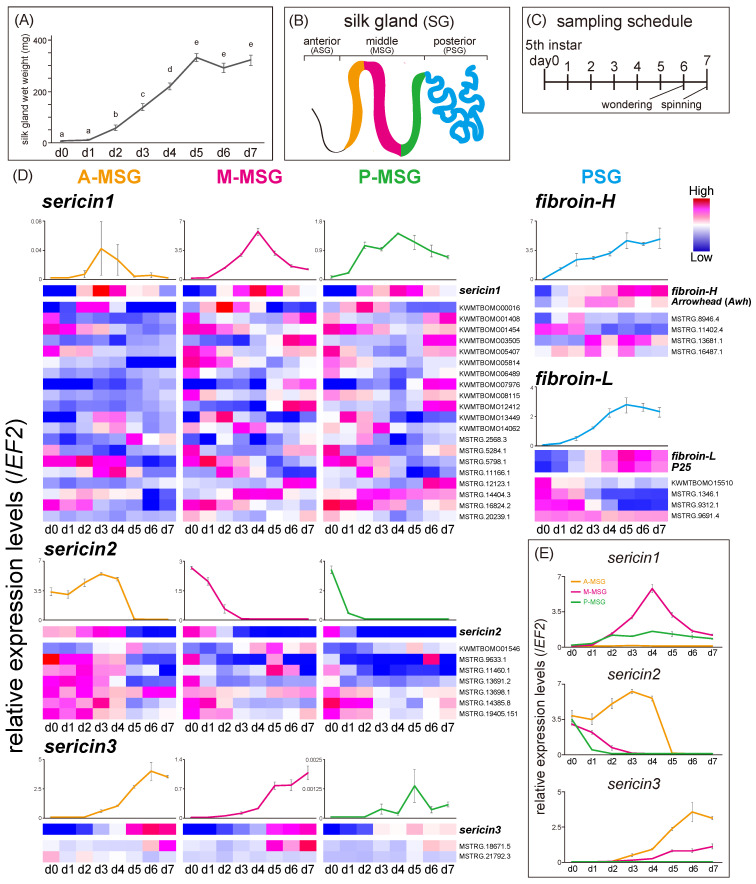
Wet weight transition of the whole SG during last instar larva (**A**). Different letters indicate significant differences in each gene (Tukey–Kramer test, *p* < 0.05). Schematic of the whole SG (**B**). Sampling schedule for qRT-PCR during last instar larva (**C**). The relative expression levels (mean ± SE, biological triplicates) of silk protein genes at each SG region during the last instar period, and a heatmap that was based on the expression of silk protein genes and their module TFs (**D**). Integrated graphs (mean ± SE, biological triplicates) showing sericin expression at each MSG region (**E**). Transcript ID is indicated on the right.

**Figure 3:**
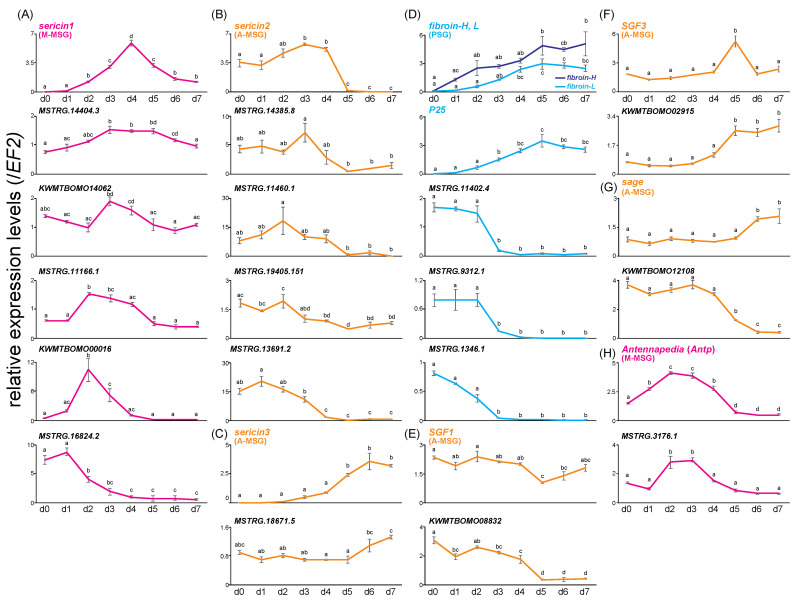
Relative expression levels (mean ± SE, biological triplicates) of each target gene and TFs at each SG region during last instar period [sericin1 (**A**), *sericin2* (**B**), *sericin3* (**C**), *fibroin*s (**D**), *SGF1* (**E**), *SGF3* (**F**), *sage* (**G**), and *Antp* (**H**)]. Different letters indicate significant differences in each gene (Tukey–Kramer test, *p* < 0.05).

It is notable that the SG developed for seven days, with the wet weight reaching a peak on the fifth day of last instar (Figure 2A). To narrow down the candidate regulatory genes, we evaluated the time-course expression pattern of TFs that were screened by co-expression network analysis in the four SG regions (A-MSG, M-MSG, P-MSG, and PSG) during the last instar period using qRT-PCR (Figure 2B–D, Figure S1A, Data S2). The expression levels of 45 TFs, showing high transcript-level TPM values among the *sericin1–3*, *SGF1*, *SGF3*, *sage*, and *Antp* modules were quantified in three regions of the MSG. *sericin1* was mainly expressed in the M-MSG and its expression level reached a peak on fourth day (Figure 2E and Figure 3A). *Antp* was also mainly expressed in the M-MSG, but its expression level reached a peak before that of *sericin1* (Figure 3H and Figure S1B). Similar to *Antp*, the expression level of five TFs belonging to the *sericin1* module (*KWMTBOMO00016*, *KWMTBOMO14062*, *MSTRG.11166.1*, *MSTRG.14404.3*, and *MSTRG.16824.2*) including homeobox domain-containing genes (Table 3) and that of a TF belonging to the *Antp* module (*MSTRG.3176.1*) reached a peak before that of *sericin1* (Figure 3A,H). *sericin2* was mainly expressed in the A-MSG, and its expression level markedly decreased on the fifth day (Figure 2E and Figure 3B). *sericin2* and four TFs (*MSTRG.11460.1*, *MSTRG.13691.2*, *MSTRG.14385.8*, and *MSTRG.19405.151*) showed similar expression patterns (Figure 3B). *sericin3* was also mainly expressed in the A-MSG, and its expression level increased over the later period (Figure 2E and Figure 3C). *MSTRG.18671.5* and *sericin3* showed similar expression patterns (Figure 3C). The expression of *SGF1* showed a similar pattern among all regions of the MSG, with the expression level decreasing on the fifth day (Figure S1B). *KWMTBOMO08832* belonging to the *SGF1* module, showed similar expression pattern to SGF1 (Figure 3E). *SGF3* was primarily expressed in the A-MSG, with its expression peaking on the fifth day (Figure 3F and Figure S1B). Although the forkhead domain-containing gene *KWMTBOMO02915* belonged to the *SGF3* module, its expression pattern was similar to that of *sericin3* (Figure 3F, Table 3). *sage* was also mainly expressed in the A-MSG, and its expression pattern was similar to that of *sericin3* (Figure 3G and Figure S1B). In contrast, *KWMTBOMO12108*, belonging to the *sage* module, showed a high expression level in the earlier period, with its expression level markedly decreasing on the fifth day. This was similar to the pattern that was exhibited by *sericin2* (Figure 3G). Furthermore, the expression levels of nine TFs belonging to the *fibroin-H* and *fibroin-L* modules were quantified in the PSG. *fibroin-H*, *fibroin-L*, and *P25* expression levels were found to be elevated through the last instar period, along with SG development (Figure 2A and Figure 3D). Although both the fibroin modules contained no TFs with expression patterns that were similar to those of *fibroin-H* and fibroin-L (Figure 2D), three TFs (*MSTRG.11402.4*, MSTRG.9312.1, and *MSTRG.1346.1*) were expressed during the earlier period, in contrast to the pattern that was exhibited by *fibroin-H* and *fibroin-L* (Figure 3D, Table 3). *Awh* was expressed through the mid-phase of the last instar period (Figure 2D, Table 3). In total, 17 TFs were eventually detected and found to be related to silk protein genes; they contained not only known regulatory factors such as the *Awh* isoform PB (*MSTRG.1346.1*) but also uncharacterized or function-unknown genes (Table 3).

## 4. Discussion

In previous studies, some genes or gene groups that are specifically expressed in the SG were identified using RNA-seq and microarray [30,31,32]. Despite this, a comprehensive screening strategy is much needed to identify the key factors that regulate silk proteins. Although *B. mori* has been previously used for co-expression network analysis [19,20], the mechanisms underlying the regulation of silk protein genes remain unclear. Therefore, in this study, we performed co-expression network as well as time-course expression analyses to identify the genes that regulate silk protein genes in *B. mori*. The co-expression network analysis was performed using an in-house program called *networkz*; consequently, 20 network modules that were related to 11 target genes were identified. The obtained TFs exhibited tissue expression patterns that were similar to those of each target gene (Figure 1), whereas, the majority of known TFs (*SGF1*, *SGF3*, *sage*, and *Antp*) formed a module that was distinct from the silk genes, respectively. Although the known TFs are co-expressed with the silk genes in the silk glands, they showed different expression patterns in other tissues, which led to the different modules. The different tissue expression patterns may be due to additional functions of these TFs which are not related with the silk gene regulation in the silk glands. These results indicated that *networkz* could successfully identify the related transcripts of each target from transcriptome data. *sericin1* and *sericin2* showed multiple modules as their mRNAs encode multiple isoforms with slightly different expression patterns at the tissue level (Table 2, Figure 1A) [4,33,34,35]. It, therefore, seems possible that diverse genes regulate *sericin1* and *sericin2* expression.

Time-course expression analysis led to the identification of 17 TFs that showed specific expression patterns and were related to target genes in the MSG and PSG during the last instar period (Figure 2 and Figure 3). The *sericin1* module contained two homeobox domain-containing genes (*MSTRG.14404.3* and *MSTRG.16824.2*), the expression of which appeared before the expression of sericin1 peaked (Figure 3A). *MSTRG.14404.3* possessed a *homothorax* (*Hth*)-like motif (Table 3). *Hth* is a known cofactor of *Antp* and is thus related to regulating sericin1 expression [4]; therefore, it appears that *MSTRG.14404.3* is also involved in *sericin1* expression regulation. Although *SGF3*, as with *SGF1*, is also involved in regulating *sericin1* expression [8,9,10], it is possible that *KWMTBOMO02915* (Figure 2D) regulates *sericin3* expression as its expression pattern was similar to that of *sericin3* in the A-MSG (Figure 3C,F). Furthermore, *KWMTBOMO02915* was already recognized as MSG-specific expression TF in a previous study [24]. The expression level of the histone superfamily gene *KWMTBOMO12108* decreased in the later period of last instar, and it was similar to that of *sericin2*. It has been reported that 20-hydroxyecdysone (20E) titer increases in the later period of last instar [36], and that 20E treatment has a repressive effect on histone gene expression [37]. Hence, it is possible that *KWMTBOMO12108* regulates *sericin2* expression via 20E titer transition. The *fibroin*s modules contained *MSTRG.11402.4*, *MSTRG.9312.1*, and *MSTRG.1346.1*, which showed high expression levels during the earlier period, in contrast to the *fibroin*s expression pattern. *MSTRG.11402.4*, MBF2 partial transcript, is reportedly involved in *fibroin-H* expression regulation [38] and is also involved in nuclear transport in the SG along with *FTZ-F1* [39]. Although *Awh* isoform PA (*KWMTBOMO00651*) and *Awh* isoform PB (*MSTRG.1346.1*) belonged to the *fibroin*s modules, their expression patterns were different during the last instar period (Figure 2D and Figure 3D). Besides, although the TFs *KWMTBOMO02915* and *KWMTBOMO12108* showed similar expression patterns with their target genes at the tissue level (Figure 1A), different expression patterns from their target genes were observed in the time-course expression (Figure 3F,G). These results suggested that when designing a screening strategy, including both co-expression network and time-course expression analyses is pivotal. As stated earlier, the TFs *MSTRG.11402.4*, *MSTRG.14404.3*, *MSTRG.1346.1*, and *KWMTBOMO02915* are known to be related to silk protein genes, while 13 novel function-unknown TFs were recognized as candidates of silk proteins regulation factor. Herein we performed time-course expression analysis to screen related TFs by qRT-PCR focusing on the candidates. Extending this approach to co-expression network analysis using RNA-seq data will help to provide insights into full extent of silk protein genes regulation.

## 5. Conclusions

In this study, silk protein regulatory genes in *B. mori* were identified using a two-step screening strategy. In the first step, 20 network modules including 91 TFs were screened by co-expression network analysis using the in-house program *networkz*, and in the second step, 17 transcripts were screened as silk protein-related genes by time-course expression analysis of the MSG and PSG during the last instar period. Since four of these TFs were already known to be related with the silk gene, we found 13 TFs as candidates for novel silk regulatory factors. As we identified both known as well as function-unknown TFs, we believe that our strategy is robust and highly sensitive to screen relative genes. Furthermore, screening results indicated that a larger number of genes than expected may be involved in silk protein regulation in *B. mori*. Functional analyses of function-unknown TFs should further our understanding of the mechanisms underlying silk protein regulation.

## Figures and Tables

**Table 1:** RNA-seq datasets using co-expression network analysis.

Series	Tissue	Strain	SRA Run ID	Replicate	Reference
RNA-seq 1	testis (TT)	o751	DRR068893-068895	3	Kikuchi et al., 2017 [21]
	fat body (FB)	o751	DRR095105-095107	3	Kobayashi et al., 2019 [23]
	midgut (MG)	o751	DRR095108-095110	3	Ichino et al., 2018 [22]
	Malpighian tubule (MT)	o751	DRR095111-095113	3	Kobayashi et al., 2019 [23]
	whole silk gland (SG)	o751	DRR095114-095116	3	Kobayashi et al., 2019 [23]
RNA-seq 2	anterior SG (ASG)	p50T	DRR186474-186476	3	Yokoi et al., 2021 [24]
	anterior middle SG (A-MSG)	p50T	DRR186477-186479	3	Yokoi et al., 2021 [24]
	middle middle SG (M-MSG)	p50T	DRR186480-186482	3	Yokoi et al., 2021 [24]
	posterior middle SG (P-MSG)	p50T	DRR186483-186485	3	Yokoi et al., 2021 [24]
	posterior SG (PSG)	p50T	DRR186486-186488	3	Yokoi et al., 2021 [24]
	fat body (FB)	p50T	DRR186489-186491	3	Yokoi et al., 2021 [24]
	midgut (MG)	p50T	DRR186492-186494	3	Yokoi et al., 2021 [24]
	Malpighian tubule (MT)	p50T	DRR186495-186497	3	Yokoi et al., 2021 [24]
	testis (TT)	p50T	DRR186498-186500	3	Yokoi et al., 2021 [24]
	ovary (OV)	p50T	DRR186501-186503	3	Yokoi et al., 2021 [24]

**Table 2:** Total transcripts and TFs in each gene module.

Target Gene	Modules	Total Transcripts	Transcription Factor
total	1022		
*sericin1*	7	565	39
*sericin2*	6	289	11
*sericin3*	1	36	2
*fibroin-H*	1	42	5 (including *Arrowhead*)
*fibroin-L*	1	80 (including *P25*)	4
*SGF1*	1	119	11
*SGF3*	1	120	6
*sage*	1	114	8
*Antennapedia*	1	126	5

**Table 3:** Domain and description of focused TFs.

Transcript ID	Module	Domain (PfamID)	Description (NCBI-nr)
KWMTBOMO00016	*sericin1*	zf-CCHC (PF00098), RT_RNaseH (PF17917), RVT_1 (PF00078), rve (PF00665)	unnamed protein product [*Plutella xylostella*]
KWMTBOMO14062	*sericin1*	zf-C2H2_4 (PF13894), PI-PLC-Y,X (PF00387, 00388), SH2 (PF00017), SH3_1 (PF00018), C2 (PF00168)	endonuclease-reverse transcriptase [*Bombyx mori*]
MSTRG.11166.1	*sericin1*	bZIP_1 (PF000170)	uncharacterized protein LOC101735428 isoform X2 [*Bombyx mori*]
MSTRG.14404.3	*sericin1*	Homeobox_KN (PF05920)	homeobox protein homothorax-like [*Bombyx mori*]
MSTRG.16824.2	*sericin1*	zf-C2HC_2 (PF13913)	homeobox protein 5 isoform X8 [*Bombyx mori*]
MSTRG.11460.1	*sericin2*	NCU-G1 (PF15065)	glycosylated lysosomal membrane protein B [*Bombyx mori*]
MSTRG.13691.2	*sericin2*	CENP-F_leu_zip (PF10473)	uncharacterized protein LOC114240082 [*Bombyx mandarina*]
MSTRG.14385.8	*sericin2*	Bromodomain (PF00439)	bromodomain adjacent to zinc finger domain protein 1A isoform X3 [*Bombyx mori*]
MSTRG.19405.151	*sericin2*	FLYWCH_zf (PF04500), BTB/POZ (PF00651)	Mod(mdg4)-heS00531 [*Bombyx mori*]
MSTRG.18671.5	*sericin3*	HSF_DNA-bind (PF00447)	heat shock factor-d isoform X4 [*Bombyx mori*]
MSTRG.11402.4	*fibroin-H*	MBF2 (PF15868)	MBF2, partial [*Bombyx mori*]
MSTRG.1346.1	*fibroin-L*	LIM (PF00412)	arrowhead PB [*Bombyx mori*]
MSTRG.9312.1	*fibroin-L*	Myb_DNA-bind_7 (PF15963)	transcription factor TFIIIB component B’’ [*Bombyx mori*]
KWMTBOMO08832	*SGF1*	zf-CCHC (PF00098), rev (PF00665), Integrase_H2C2 (PF17921), Asp_protease_2 (PF13650)	uncharacterized protein LOC114250529 isoform X1 [*Bombyx mandarina*]
KWMTBOMO02915	*SGF3*	Forkhead (PF00250)	fork head domain-containing protein FD4 [*Bombyx mori*]
KWMTBOMO12108	*sage*	Histone (PF00125), CBFD_NFYB_HMF (PF00808)	nuclear Y/CCAAT-box binding factor C subunit NF/YC isoform X1 [*Bombyx mori*]
MSTRG.3176.1	*Antennapedia*	MTABC_N (PF16185)	transcriptional regulator ATRX homolog [*Bombyx mandarina*]

## Data Availability

Not applicable.

## Supplementary Materials

The following materials are available online at figshare. Figure S1: The relative expression levels of known regulatory factors at each SG region during the last instar period, and a heatmap that was based on the expression of known regulatory factors and their module TFs. (doi:10.6084/m9.figshare.17141888). Table S1: Primer sets that were used in this paper (doi:10.6084/m9.figshare.17141969). Data S1: Output raw data of co-expression network analysis using *networkz* (doi:10.6084/m9.figshare.17427359). Data S2: Relative expression levels of all tested transcripts (doi:10.6084/m9.figshare.17427362).
